# How Therapists Use Visualizations of Upper Limb Movement Information From Stroke Patients: A Qualitative Study With Simulated Information

**DOI:** 10.2196/rehab.6182

**Published:** 2016-10-05

**Authors:** Bernd Ploderer, Justin Fong, Marlena Klaic, Siddharth Nair, Frank Vetere, L. Eduardo Cofré Lizama, Mary Pauline Galea

**Affiliations:** ^1^School of Electrical Engineering and Computer ScienceQueensland University of TechnologyBrisbaneAustralia; ^2^Microsoft Research Centre for Social Natural User InterfacesThe University of MelbourneParkvilleAustralia; ^3^Department of Mechanical EngineeringThe University of MelbourneParkvilleAustralia; ^4^The Royal Melbourne HospitalParkvilleAustralia; ^5^Department of Medicine (Royal Melbourne Hospital)The University of MelbourneParkvilleAustralia

**Keywords:** stroke, upper-limb rehabilitation, therapy, information visualization, dashboard, wearable technology

## Abstract

**Background:**

Stroke is a leading cause of disability worldwide, with upper limb deficits affecting an estimated 30% to 60% of survivors. The effectiveness of upper limb rehabilitation relies on numerous factors, particularly patient compliance to home programs and exercises set by therapists. However, therapists lack objective information about their patients’ adherence to rehabilitation exercises as well as other uses of the affected arm and hand in everyday life outside the clinic. We developed a system that consists of wearable sensor technology to monitor a patient’s arm movement and a Web-based dashboard to visualize this information for therapists.

**Objective:**

The aim of our study was to evaluate how therapists use upper limb movement information visualized on a dashboard to support the rehabilitation process.

**Methods:**

An interactive dashboard prototype with simulated movement information was created and evaluated through a user-centered design process with therapists (N=8) at a rehabilitation clinic. Data were collected through observations of therapists interacting with an interactive dashboard prototype, think-aloud data, and interviews. Data were analyzed qualitatively through thematic analysis.

**Results:**

Therapists use visualizations of upper limb information in the following ways: (1) to obtain objective data of patients’ activity levels, exercise, and neglect outside the clinic, (2) to engage patients in the rehabilitation process through education, motivation, and discussion of experiences with activities of daily living, and (3) to engage with other clinicians and researchers based on objective data. A major limitation is the lack of contextual data, which is needed by therapists to discern how movement data visualized on the dashboard relate to activities of daily living.

**Conclusions:**

Upper limb information captured through wearable devices provides novel insights for therapists and helps to engage patients and other clinicians in therapy. Consideration needs to be given to the collection and visualization of contextual information to provide meaningful insights into patient engagement in activities of daily living. These findings open the door for further work to develop a fully functioning system and to trial it with patients and clinicians during therapy.

## Introduction

Stroke is the leading cause of acquired adult disability in high-income countries [1], with upper limb deficits affecting an estimated 30% to 60% of survivors [2,3]. Stroke causes damage within the brain that, when affecting somatosensory circuitry, lead to difficulties sensing and controlling movement of the body’s contralateral side. Due to these limitations, stroke patients tend to reduce the utilization of the affected limb, which may cause muscle shortening and weakness, thus further compromising arm functionality [4]. As a result, performance in basic activities of daily living (ADL) such as eating, bathing, and dressing can be heavily affected, impacting on a patient’s independence, social engagement, quality of life, and well-being [5].

Therapists (occupational therapists and physiotherapists) deliver effective upper limb rehabilitation interventions in hospitals. Interventions generally start by setting goals that target meaningful activities (eg, use of cutlery), functional movements (eg, grasp and retrieve objects), or specific impairments (eg, muscle weakness). Training is often task-specific and involves practicing tasks relevant to daily life. Along with this training, therapists employ a variety of techniques to support rehabilitation, such as mirror therapy, muscle electrical stimulation, strength training, stretching and positioning, mental practice, robotics, and virtual reality applications [4,6-8].

Since therapy time is limited, the use of the affected arm in between sessions is crucial for enhancing functional outcomes. Therapists generally prepare daily exercise routines considering a patient’s personal goals, or they utilize constraint-induced movement therapy to encourage patients’ use of the affected arm in daily life [4]. Although the use of activity diaries such as the Motor Activity Log (MAL) allow determining compliance with therapy when not in the clinic, these are subject to various biases including the ability and motivation of patients and caregivers to provide accurate information [9]. The lack of objective information is particularly concerning because adherence to rehabilitation programs at home is often low due to lack of motivation, musculoskeletal issues, and fatigue [10].

Wearable sensor technology offers potential to provide therapists with objective information about a patient’s arm movement in everyday life. Specifically, inertial measurement units (IMUs) appear promising, because these sensors can be embedded in wristbands, gloves, or garments, and thereby track changes in the acceleration and orientation of the affected arm. Various studies in controlled settings show that IMUs can track arm, hand, and finger movements [11-14]. This line of research is typically focused on technical challenges (ie, the accuracy of motion tracking [12,15]), reliability of tracking over long periods of time [16], wearability for patients [17], and the processing of metrics from sensor data [18]. While all of these issues are important to realize the potential of wearable sensor technology, to date there has been little consideration for the needs of therapists and whether this information is useful for the rehabilitation process.

The aim of this research is to explore the information needs of therapists in order to help them understand how patients use their arm in everyday life in between rehabilitation sessions. In particular, this research seeks to address how therapists use visualizations of upper limb information presented on a dashboard to support therapy. A dashboard in this sense refers to a visual display of information on a computer screen. Similar to a car dashboard, the information on a digital dashboard needs to be compact to be monitored at a glance, to help people achieve one or more objectives [19]. Since neither wearable sensors nor dashboards are readily available, we conducted a design-driven investigation where we built a dashboard prototype that visualizes arm movement information, and we evaluated this Web-based prototype in a qualitative study with therapists. Based on a qualitative analysis we discuss the potential uses of these visualizations and identify areas for improvement.

## Methods

### Dashboard Design Process

The dashboard design process is part of a larger research project into the development of a system to monitor upper limb movement of stroke patients in everyday life. The envisioned system consists of (1) wearable sensor technology that patients wear on their arm over several weeks to monitor upper limb data in everyday life; and (2) a dashboard to present the sensor data to therapists for use in consultations with patients.

A wearable sensor prototype has been evaluated in a movement laboratory to establish the feasibility of this approach [20]. The prototype captures motion of the arm through IMUs placed at the wrist, above the elbow, and at the shoulder. From these sensors, motions in three degrees of freedom in the shoulder (adduction/adduction, flexion/extension, internal/external rotation), one in the elbow (flexion/extension), and one in the wrist (pronation/supination) can be calculated. The current system is not capable of capturing wrist extension or finger movements. The project team is now working on a sensor prototype that is comfortable to wear and robust enough for use in everyday life.

We designed a dashboard prototype that visualizes sensor data to support therapists in their consultations with patients. The prototype was created through a user-centered design process, a standard approach in the field of human-computer interaction, to ensure that the dashboard that is being developed meets the needs of users [19,21]. The design process started with informal interviews with 3 occupational therapists (OTs) to understand the problems faced by therapists and the need for objective information. Based on these insights, 3 rounds of design workshops were conducted to generate and review ideas for information and visualizations that could be useful to support the work of therapists. These workshops involved 2 OTs, 1 physiotherapist, 2 mechanical engineers, 2 experts on wearable technology, and 2 interaction design researchers. As is common in a user-centered design process [22], ideas were initially sketched on paper for review and discussion. For the second and third workshops these sketches were refined as paper prototypes and digital prototypes. The final dashboard prototype was built with the prototyping software Axure, which supports the implementation of interactive Web-based prototypes without requiring software development skills. The strengths of such a prototyping approach are that they capture the key ideas of the entire team, allow quick evaluation and iteration, and facilitate discussion about relevant information and visualizations before effort is spent on developing the actual software [22,23].

### Dashboard Prototype

We developed an interactive dashboard prototype to gather feedback from therapists on the usefulness of various upper limb visualizations before a fully functioning system is implemented. As illustrated in the following figures, the prototype was designed in a sketchy manner to invite feedback, and to avoid giving the impression that this was a fully functioning website.

The dashboard prototype evaluated in this study contained upper limb movement information for each patient (Textbox 1).

This information was based on interviews and design workshops with therapists, as well as related work on kinematic measures for upper limb movements [18]. Related work shows that inertial sensors can provide information on the amount of arm movement and time spent using the arm in daily life [24]. Quality of movement and range of motion (ROM) are typically generated through robotic technologies or opto-electronic systems [18]. These systems can provide more precise measurements than inertial sensors, but they rely on a controlled environment and hence are not readily available for daily life use.

Part of the information displayed on the website was based on sensor data collected in a movement laboratory [20]. We created additional fictional information in consultation with therapists to ensure that the information presented on the dashboard is complete and realistic for a stroke patient.

The following figures show how this information was presented on the dashboard through 5 screens, which support different views and analysis of the various data.

#### Overview Page

**Figure 1:**
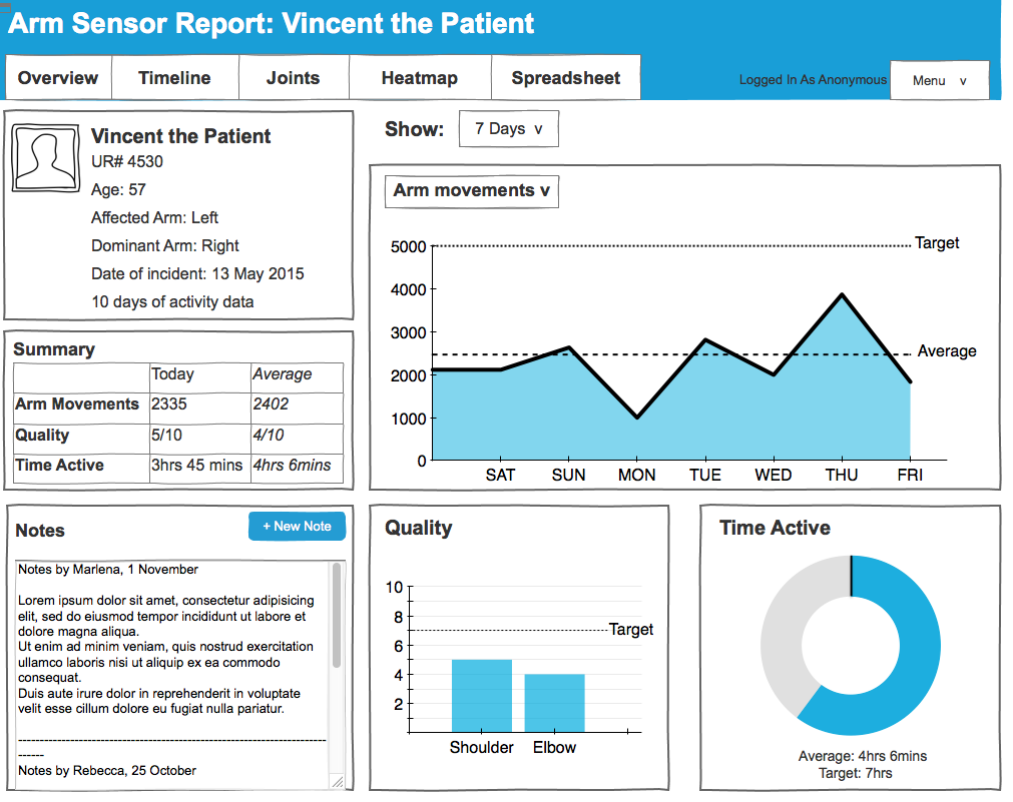
Screenshot of the overview page.

The first page provides an overview of a patient’s upper limb information (Figure 1). It includes a brief patient profile, showing age, affected arm, dominant arm, and date of incident. An overview is provided of key movement information, including a tabular summary of number of movements overall, quality of movement, and time active. The therapists in the design workshops wanted both information about averages and for particular time periods. Furthermore, a timeline shows the number of movements over the last week, and the quality of movement on a scale from 1 (low quality) to 10 (high quality). The visualizations here were inspired by related work [19] and commercial dashboards of activity trackers (eg, Fitbit, Jawbone Up). Therapists can add notes. This is important as patients are usually seen by multiple therapists in the course of their therapy.

**Upper limb information for each patient.**

1. Amount of arm movement, counting movements for each degree of freedom.

2. Time spent using the arm.

3. Quality of movement (as indicated by compensatory movements, speed, and smoothness), on a scale from 1 to 10.

4. Range of motion (ROM) for each degree of freedom.

5. A list of the above information for each detected movement.

#### Timeline Page

**Figure 2:**
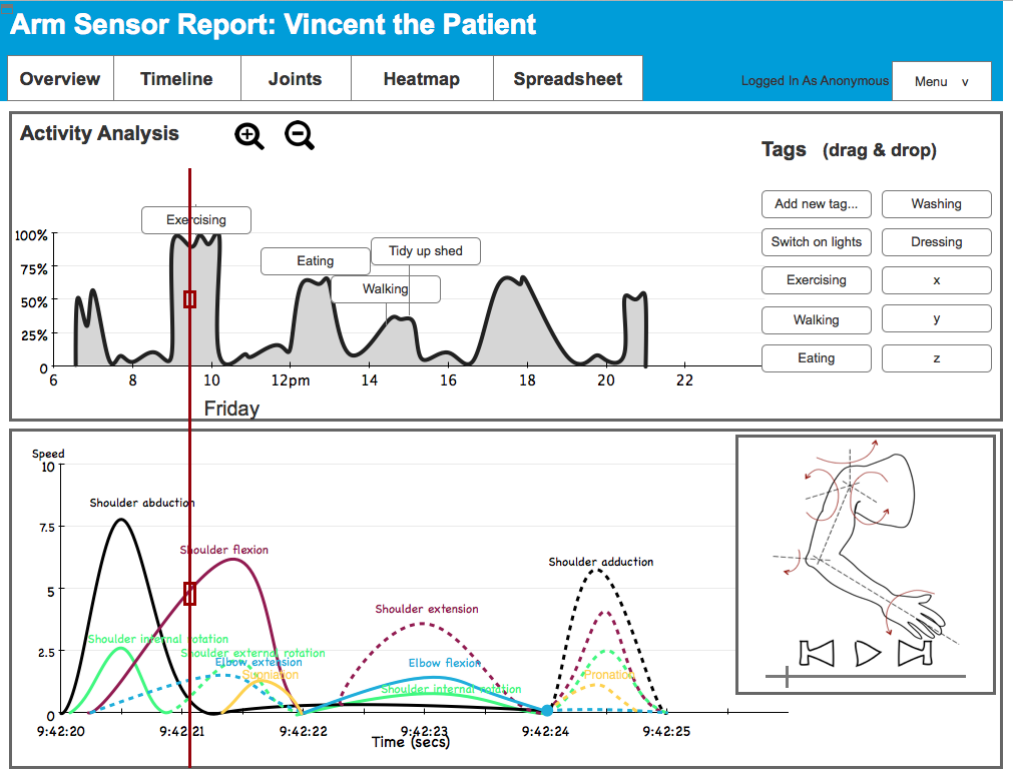
Screenshot of the timeline page.

The timeline page, which provides detailed movement information at two different time scales is shown in Figure 2. The timeline on the top presents movement patterns over long periods of time, from several hours to several days. The data presented here shows the level of activity, for example, 50% means that the arm is moved for 5 minutes during a 10- minute window. This information was included to provide therapists with a quick snapshot of how active patients are throughout a day. Therapists can annotate this data by dragging and dropping tags like “exercising” and “eating” to the activity timeline.

The timeline on the bottom of the page presents movement for each degree of freedom over several seconds. The red progress bar connects the two time lines. This information was included so that therapists can explore movement in more detail and obtain insights into the quality of movement. For example, they can select a data point in the activity timeline (on top of the page) from a period of exercising, and on the bottom of the page they can see how the exercise was performed (eg, whether the movement was initiated by abducting from the shoulder which would indicate a compensatory movement). A media player (bottom right) shows arm position and movement corresponding to the progress bar on the time line to visualize how the arm moves to aid with this analysis.

#### Joints Page

**Figure 3:**
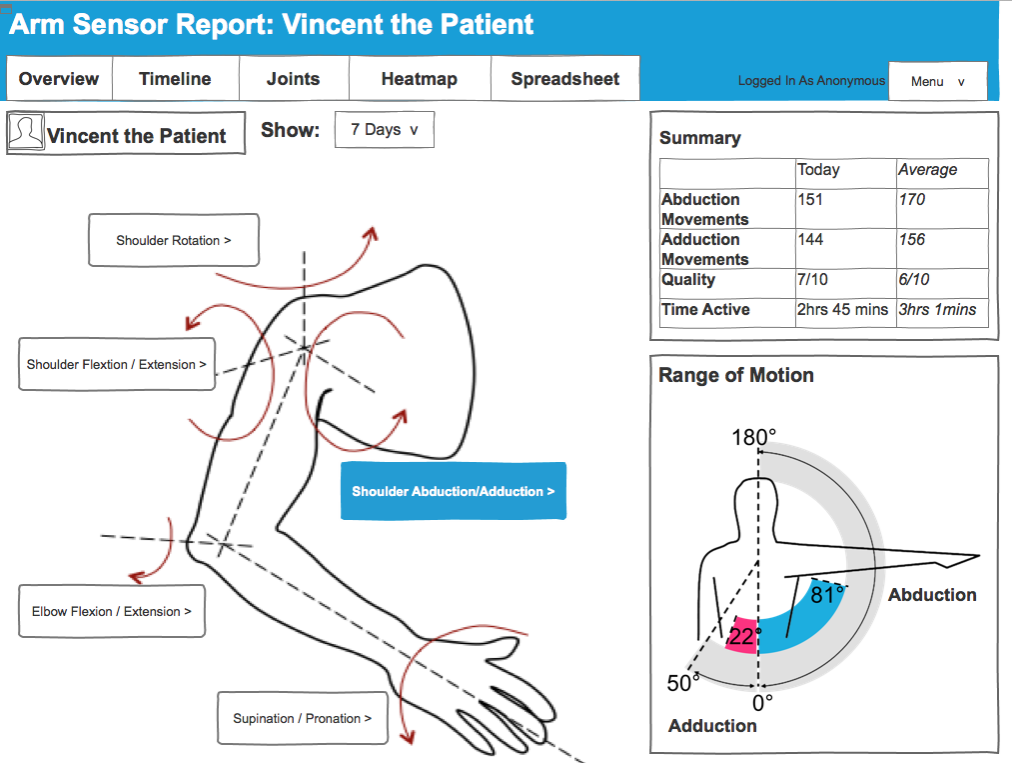
Screenshot of the joints page.

The joint-based visualization illustrated in Figure 3 structures movement information around the entire arm. Therapists can click on a particular plane of movement in each joint (eg, shoulder abduction/adduction) to access a summary of a number of movements, quality, time active, and active ROM for the selected movement. Inspired by related work [25], the ROM is further illustrated for the selected joint through an avatar that visualizes the ROM achieved by the patient in daily life compared with the maximum ROM possible for this type of movement. This page was developed during the design workshops to show patients how the information collected through sensors relates to the different types of upper limb movement.

#### Heatmap Page

**Figure 4:**
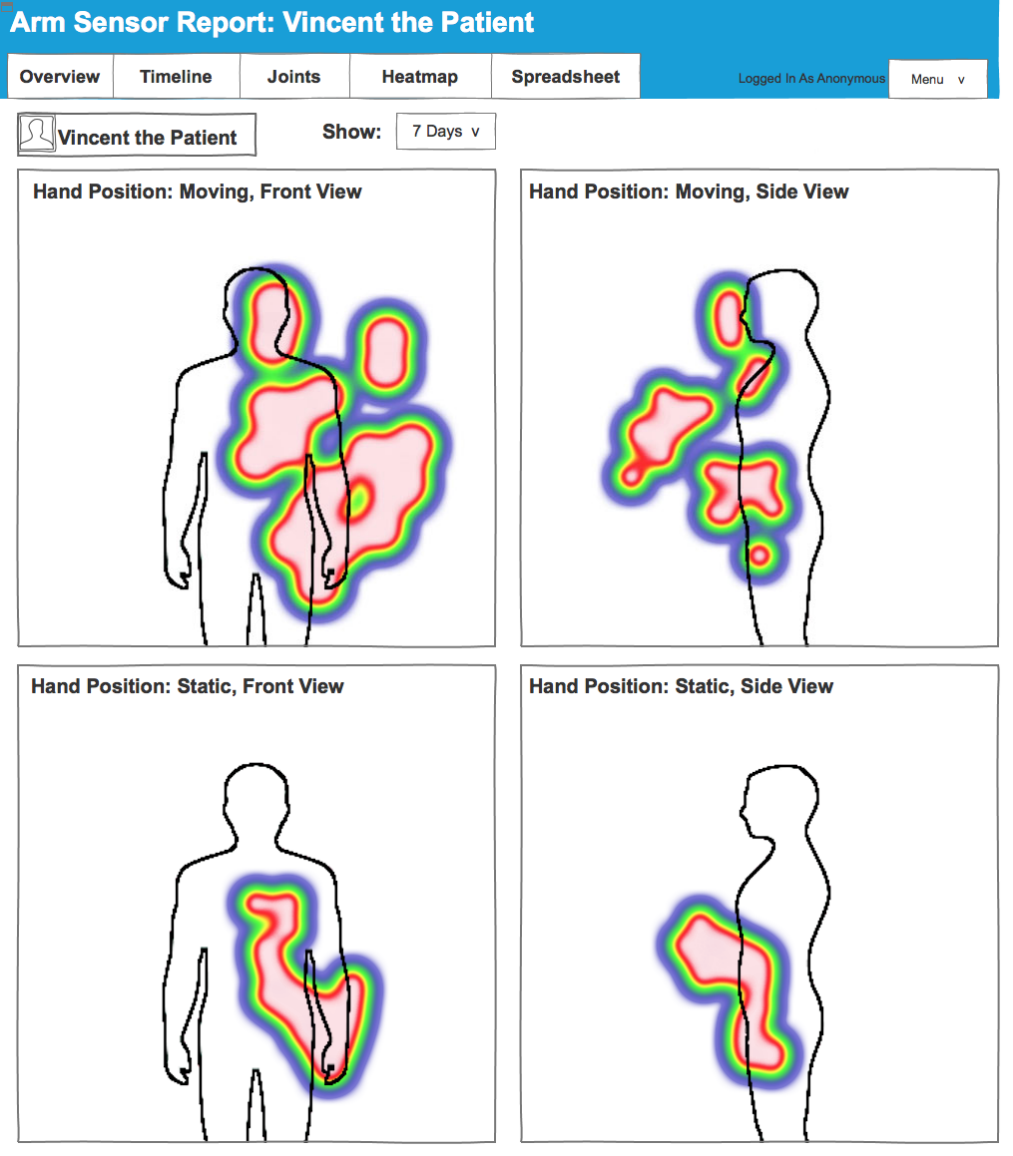
Screenshot of the heatmap page.

Figure 4 presents the heatmap page, which shows common movement (top) and common static positions (bottom) of the affected hand over the last 7 days. Areas in red show the most common movements or positions, where green and blue indicate some movement or positioning, whereas white indicates areas which were not reached by the hand in the 7-day period. The front view (left) shows whether the hand has crossed the midline, whereas the side view indicates whether patient have the capability to reach forward. Heatmaps are incorporated in the dashboard because therapists and patients are already familiar with this type of visualization from computer-based therapy games (AbleX system) used in the hospital.

#### Spreadsheet Page

**Figure 5:**
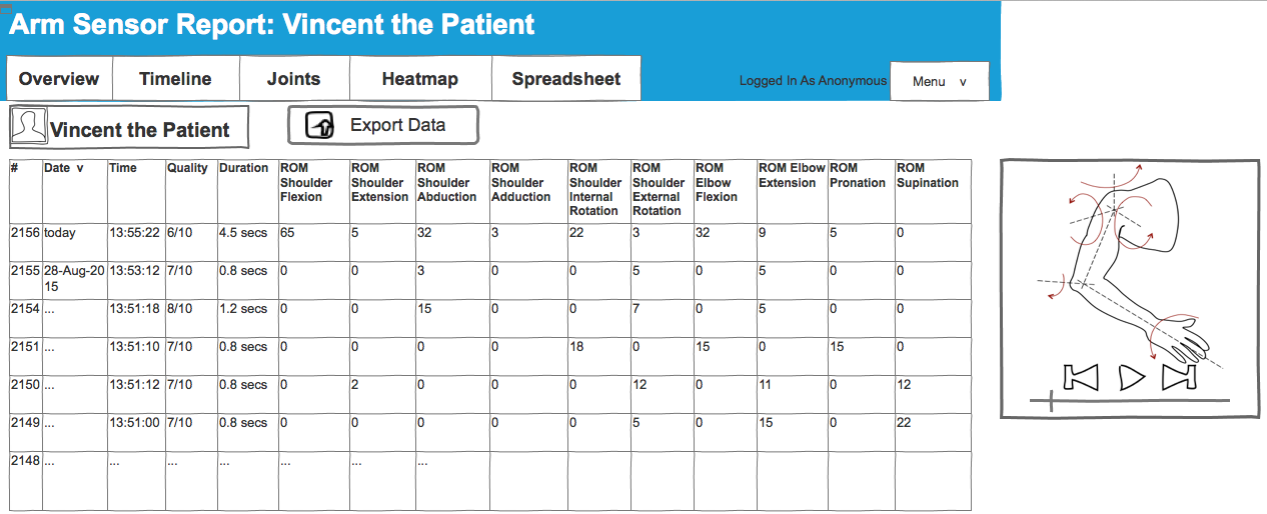
Screenshot of the spreadsheet page. ROM: range of motion.

Figure 5 shows the spreadsheet, which allows therapists to inspect all movements captured by the sensor and to sort them by time, quality, duration, and range of motion. A media player can be used to illustrate the arm movement selected in the spreadsheet. The data can be exported for further analysis (eg, for research into the effectiveness of interventions). This page was included during the design workshops to provide support detailed analysis of movements for therapists engaged in research activities.

### Study Participants

We recruited 8 therapists (all female) to evaluate the dashboard prototype. Participants were recruited through the Royal Melbourne Hospital, Australia. All therapists were actively engaged in upper limb therapy with patients with neurological conditions including stroke, multiple sclerosis, traumatic brain injuries, and Parkinson’s disease. Their clinical experience ranged from 3 months to 12 years. Five therapists worked predominantly with acute patients (within the first few weeks after presenting to hospital) and 3 therapists worked with chronic patients (ranging from several weeks to several years after a stroke). These 8 therapists had not been involved in the design process. They were recruited for the evaluation to provide unbiased feedback on the dashboard. Book vouchers were offered to participants for their time and involvement in the dashboard evaluation.

### Dashboard Evaluation

A qualitative evaluation was conducted to explore how therapists would use the information presented and visualized on the dashboard. The evaluations took place in a meeting room at the hospital and lasted 60 minutes per therapist. Ethics approval was obtained through the University of Melbourne (#1545866).

The evaluation followed a standard procedure. First, a background interview was conducted to learn about upper limb rehabilitation practices and the information therapists desire about their patients. Second, we conducted observations of therapists exploring each of the 5 dashboard pages. The therapists were instructed to think aloud in order to get a better understanding about their impressions of each visualization on the website and any questions or expectations that they may have. Finally, through a semi-structured interview, the therapists were asked to compare and rate the 5 visualizations in terms of usefulness for their work with stroke patients. These ratings were used as prompts to discuss how the dashboard could be integrated with their current work practices and the potential impact on improving rehabilitation outcomes.

Each evaluation was audio-recorded and transcribed for later analysis. The examination of the dashboard was also screen-recorded with input from a webcam to capture facial expression of participants as they interacted with the website.

The data were analyzed qualitatively, following a thematic analysis approach [26]. The authors read through all transcripts and coded the data to identify the various uses for each visualization as well as areas for improvement. Data were coded by the authors (BP, JF, SN) through SaturateApp, a Web-based tool for collaborative qualitative analysis. In total, 249 codes were generated about the uses for the 5 dashboard pages, 35 codes about ranking the different visualizations according to their potential usefulness, and 55 codes about the usefulness of the dashboard as a whole. In consultation with the research team these codes were collated into 3 themes that describe the uses of the dashboard and 1 theme about a major limitation in using the system, which are presented next.

## Results

### Theme 1: Objective Data About Activity Levels, Exercise, and Neglect

The main use of the dashboard is to obtain objective patient data. Therapists can glance at the dashboard before or during consultations to assess how patients engage their upper limb outside the clinic including how actively they engage the affected limb, their adherence to exercise regimens, and possible neglect of the affected limb.

The overview page was preferred by 63% (5/8) of therapists to assess the activity levels of patients outside the clinic. The overview page provides a quick snapshot of the patient’s activity levels through visualizations of the number of movements performed over a week, the average quality of these movements, and the time spent active for each day. A simple timeline showing movements performed over a week offers therapists a quick glance of days when their patients performed well and when their patients did not reach their target levels.

A lot of patients will try really hard today, and then tomorrow they really suffer, and then the next day they will probably do somewhere in between, and then two days later they will be like "oh I haven’t done my exercises very much." And educating a patient around that when you’ve got hard data spike is really valuable.

OT8

The timeline page was preferred to assess whether patients adhered to the prescribed exercise regimens. The first visualization on this page shows the times and the intensity of arm activities over several days. Therapists used this information to infer activities based on time (eg, eating), duration (eg, exercise), or through conversation with patients. Some patients keep exercise diaries that therapists can use to compare with the timeline data. The timeline supports tagging, meaning that therapists can manually annotate events on the timeline with labels such as exercising and eating. It is important to note that the second timeline on the bottom of this page was not considered useful. This timeline would support analysis of movements for each degree of freedom over several seconds, for example, to inspect how patients perform an exercise. However, therapists commented that they would not have the time to analyze the data in this way.

If you’re worried that he’s not doing his exercises, or he’s not incorporating his hand when he’s eating, well this would somewhat tell you whether there’s a flat line or whether there are moments of activity.

OT5

We could get them to keep a diary or something like that, and when they come then sit down with their diary. I like the idea there is some sort of analysis of the activities even though you have to look at each patient and think about if it's accurate or not.

OT3

We work on a busy rehab ward, would we actually come back to this and really analyze [the second timeline on the bottom of the page] to every five seconds?

OT5

Finally, therapists found the heatmaps useful to assess patients with very low levels of mobility and patients with hemispatial neglect, who have difficulty attending to one side of space. The heatmaps indicate where the hand is resting, and can be used to identify whether the hand is resting in a “natural” position. The heatmaps also show whether the hand of the patient crosses the midline of their body. This indicates attendance to the neglected side in neglect patients, and it shows an increased range of activities of daily living that a patient is able to perform.

You want to know when they’re sitting particularly the ones that have neglect, do they just leave it dangling down here, or are they positioning it in an appropriate way? I like that. It’s good.

OT4

If you can cross midline and do stuff you are getting better plasticity showing but you’re also functionally significantly more independent than if you can only work here.

OT8

### Theme 2: Engage Patients to Learn About Therapy, Provide Motivation, and Reflect on Progress

A second area of use for the dashboard is to engage patients in a dialogue about the data to become more actively involved in the rehabilitation process. Therapists and patients can collaboratively examine the data presented on the dashboard to foster motivation and to inquire how patients cope in their everyday life.

Particularly the timeline data and the tagging feature invited opportunities for therapists to engage their patients to learn more about exercise and other activities. Therapists can use the data to inquire about how well patients cope with the exercise programs that they have been given. Therapists may also use peaks and troughs in the timeline data to ask more broadly about the well-being of their patients in daily life.

I'd sit down with the patient and ask what they were doing between 8am and 10am on Friday, and they say they went to the gym. So I put in exercise.

OT3

Are they coping with what I've given them? If they're not doing their exercises, why?

OT7

Furthermore, therapists used the dashboard (ie, the ROM presented on the joints page) to educate and motivate patients. Therapists wanted to use the data to teach patients how the arm works, what their capabilities are, and to discuss how they are progressing. Improvements in the ROM are not always visible to patients and therapists, and therapists typically do not have the time to assess ROM with a goniometer in each therapy session. Seeing progress in ROM through the joints page, however, was useful to see how patients progress over the course of a therapy as well as to detect discrepancies between how patients perform in therapy and how they perform at home. ROM is also an important indicator of the activities of daily living that a patient is able to perform. For example, activities like feeding require a certain range of motion to extend the elbow and to supinate at the wrist. Hence, based on the information about the ROM displayed in the joints section therapists and patients discuss their goals.

It would be nice to be able to give the patients this feedback and show them visually how they are doing, and be able to say "this is where we want you to be. This is your target for the next 2 weeks." And then you could be pushing that target out as they improve.

OT1

It’s going to help me visualize their movement. If I know that they can only get to 181° for the certain task that they pick during the day, you can sort of know how they would perform it. And it also gives us goals to work on, to increase that range of movement.

OT4

Finally, therapists found the visualizations on the overview page and the heatmaps useful to engage patients in discussion about the rehabilitation progress. The overview page provides simple visualizations of the number of movements carried out by a patient that can illustrate improvements and thereby motivate patients to adhere to their exercise regimens and goals. Heatmaps, on the other hand, are useful to engage patients in discussions about which areas they need to target when moving their arm. Some therapists emphasized that the dashboard provides a useful, additional voice to the therapy that motivates patients.

I use that in two senses - to provide patients with motivation and say they've improved a little more this week; and the flip side is if they're not improving I provide realistic feedback so in three weeks’ time, when I discharge them from the service and they're ‘my arm hasn't improved’, it's not a shock to them.

OT3

If it [the heatmap] was all just red by his body I could talk to him about it’s really important to let that arm sit down and extend the elbow to involve it one day in swinging while he’s walking.

OT2

I think it's quite motivating for patients. It's not just me speaking to them.

OT7

### Theme 3: Engage With Other Clinicians and Researchers Based on Objective Data

The information presented on the dashboard can also be useful beyond the interactions between a therapist and a patient during therapy. It provides therapists with objective data to advocate for patients in interactions with other clinicians. For example, providing evidence about improvements in the range of motion in everyday life can help to persuade other clinicians about the importance of upper limb therapy. Objective data is useful here, because therapists often rely on subjective judgments about a patient’s ability to participate in activities of daily living, and such judgments are difficult to translate between health professionals. Both forms of evidence are important to advocate for patients to receive adequate resources required for rehabilitation.

Other therapists, your physio colleagues, or your doctors, they can actually see that the patient’s arm movement is improving. So if they started off with no movement at the shoulder whatsoever, but three weeks down the track they’re actually generating some active movement.

OT5

Being able to show other team members what movements are improving, and the doctors as well, it would be awesome to take this data to a team meeting and to show how much a patient has improved from a movement point of view. Because often what we are doing is advocate for rehab. And not every patient gets the rehab. If we can show to the team that they made all these improvements in terms of arm function, our case would be so much stronger.

OT1

Finally, the information available through the dashboard provides opportunities for research into the effectiveness of rehabilitation services provided at the clinic. The spreadsheet page allows therapists to sort data by time, duration, and quality to support detailed analysis of the motions performed by individual patients. While the spreadsheet page was not considered useful for therapy, being able to export this data was seen as useful for further therapists engaged in research activities in order to assess the effectiveness of interventions across different patients.

Your spreadsheet is only helpful for data analysis and research, which I think is a great thing to have incorporated but there’s only going to be a small group of people that would utilize that.

OT8

### Theme 4: Contextual Information is Critical to Analyze Movement Data

A major limitation is the lack of contextual information presented across the different dashboard pages. The different dashboard pages presented various movement data (number, range, duration, quality of movement). However, a recurring discussion point with therapists was the lack of contextual information to understand the significance of these movements in daily life.

First, the lack of contextual information was evident in discussions of the quality ratings. The quality rating was displayed on the overview page as an average value between 1 and 10 for all the movements performed over the course of a day, thus allowing the therapists to see trends in the data over several days and weeks. The therapists confirmed the findings from study 1 that information about the quality of the movements outside the clinic is critical, for some even more so than the number of movements. However, while the therapists desired a quality score, they also felt that in order to truly judge the quality of a movement they would have to see their patient making the movement. This is because the quality of a movement is dependent on its purpose in a particular context. For example, lifting the shoulder and shoulder abduction are often used as indicators for low quality movements, because many stroke patients use these movements to compensate for difficulties in reaching forward, or involuntarily abduct the shoulder when intending to reach forward. However, in certain contexts lifting the shoulder and abduction can be desirable and indicative of a normal, high quality movement, which cannot be distinguished by the system.

It is important that they do their activities well, not just a lot.

OT1

I have some questions about measuring this one, quality. This doesn't have any way to determine the movements are of quality and whether they're normal or not, it's just detecting [motion] - for some tasks a quality movement would be to abduct your arm like this so you bring your hand up to do your hair, and for reaching to abduct your arm isn't a normal movement. So if you're able to measure abduction but then you're not able to know what the task is they're doing, how do you determine whether that's a quality movement for that task?

OT3

Second, the lack of contextual information was evident in discussions about the timeline page. Based on the dashboard alone therapists cannot know if a movement constitutes an exercise activity, if the patient is engaging in an activity of daily living like eating, if the arm is swinging while walking, or if the arm is moved by a caretaker who helps the patient get dressed. The timeline presents some contextual information through the time of the day when movements are performed, which can indicate that a patient is eating or washing. However, the precise nature of the activity needs to be confirmed in conversation with a patient.

I find it really hard because you don’t know what they’re doing when they’re doing this movement. Like I could be walking, going like this, and that’s going to be counting the movement of every joint whereas it’s not specifically functional.

OT4

The lack of contextual information provides opportunities for encouraging participation by patients. On the one hand, therapists commented that some patients would be interested in collecting contextual information, for example, through a mobile app that would help them to diarize events. On the other hand, the lack of contextual information provides an opportunity for increased patient participation during consultations through dialogue about the data. Patients contribute their lived experience and therapists their domain knowledge to collectively interpret the data.

For patients that were more technologically savvy you could do something like getting them to write down at the end of the day what it is that they’ve done, and I think with some of the more cognitively impaired or older patients, that would be really difficult for them to reflect back on "what did I do yesterday at different times of the day?" So that’s why I think having something to support it, like a time use diary or a written diary or a phone app, would be really useful.

OT6

We can actually show them the days that they are doing better, and actually talk about, let’s say "Monday wasn’t so good", maybe they had a lot of scans and investigations. Or maybe they had a really bad day and didn’t want to do their rehab.

OT1

## Discussion

### Principal Findings

This research identified core principles for the visualization of information collected through wearable sensor technologies for use by occupational therapists.

Dashboards provide objective data for therapists about the activities of patients outside the clinic. This is important because prior work shows that the quality of subjective data through retrospective recall and exercise diaries is limited, and it relies on patients who are motivated and have adequate cognition [9]. Hence, data from wearable devices presented on the dashboard can verify subjective accounts from patients through objective data about activity levels in between therapy sessions, exercises performed at home, and attendance to the neglected side of the body.

In accessing objective data, therapists emphasized the importance of getting an overview, over being able to see details. In line with the principal idea of a dashboard [19], the overview needs to provide a quick glance of the patient data. This overview needs to support comparison between different timescales, from several hours to several weeks, and between different joint movements (eg, to compare shoulder abduction with shoulder flexion). Unlike in other domains [27], the therapists expressed that they would not have time to inspect details of individual movements or outliers in the data, because it would take time away from working hands-on with patients. Hence the spreadsheet and the detailed timeline to analyze movements over several seconds were seen as superfluous.

Visualizations need to engage patients in the therapy process. In particular, visualizations play an important role in discussing progress, motivating patients, and prompting reflection about exercises and activities of daily living performed in their own homes. Timeline visualizations were useful to discuss progress with patients. Heatmaps were useful to present spatial information about common positions and postures of the arm for reflection with patients. This is important to foster patient participation and motivation to achieve positive rehabilitation outcomes [28].

Visualizations and objective data are important to help therapists advocate on behalf of their patients in discussions with other clinicians. The work of therapists depends to a large extent on subjective judgments about a patient’s ability to engage in activities of daily living. Hence, having objective movement data captured in daily life provides an objective indicator of a patient’s capabilities that therapists can use in discussions with other clinicians.

Contextual information is critical to analyze the information visualized on the dashboard. The lack of contextual information was raised as a key limitation because the therapists wanted to understand how much patients use their affected upper limb in daily life outside therapy (eg, to exercise, eat, or dress themselves). There was a disparity between the generally hands-on work of therapists, where they can touch and observe patients and understand the intentions of their actions, and the visualizations generated from sensor data that were disembodied and lacked references to the settings in which movements occur. Prior work on clinicians interpreting sensor data from patients with Parkinson’s disease [29] and multiple sclerosis [30] highlights similar challenges in interpreting sensor data where therapists find it difficult to interpret sensor data in the absence of the patient, even though these studies [29,30] used sensors for short assessments in clinical settings, rather than to collect data over days and weeks in real-life. Health data are often not self-evident, and additional work is required to make sense of the data and to apply it in practice [29,31]. However contextual information is particularly important for therapists to interpret body movement, including understanding how movements relate to activities of daily living ranging from personal and domestic tasks, to community, employment, leisure, and recreational activities [32]. Hence, subsequent phases of this project will explore how contextual information can be gathered, such as through sensors embedded in objects and places that indicate activities (like sensors embedded in cutlery to indicate eating), or through mobile apps that allow patients or their caretakers to annotate movement information with pictures or personal notes about daily life activities. Furthermore, we seek to investigate to what extent the revised dashboard can elicit contextual information through dialogue between patients and therapists.

**Figure 6:**
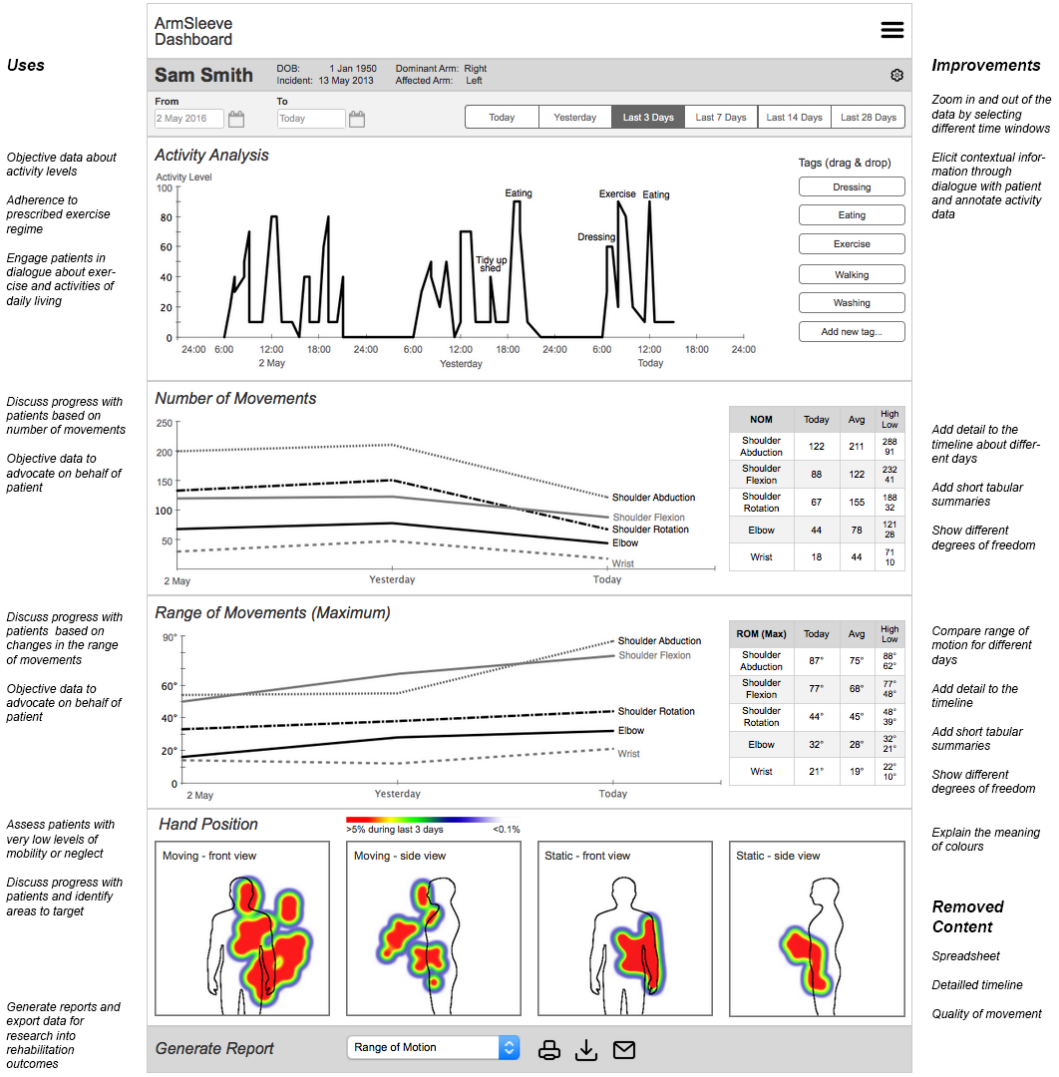
Revised dashboard design based on the findings from this study. The annotations on the left side show how the new design maintains the key features that the therapists found useful. The annotations on the right side highlight changes to the design.

Figure 6 summarizes the findings through a revised dashboard design. Based on the results presented above we combined the most useful elements of the 5 original dashboard pages into a design that fits on a single page to support meaningful comparison and minimize time spent navigating the dashboard. The annotations to Figure 6 summarize the key findings about the uses of the dashboard (obtain objective data, and to engage patients and clinicians) and the areas identified for improvement (capture contextual information, changes to enhance the clarity of the information presented, and content omitted due to lack of use).

### Limitations

The main limitation of this study lies in the ecological validity. The findings of this study provide rich insights into the potential uses of a dashboard to support upper limb therapy. However, evaluations in a laboratory or simulated setting do not allow for evaluation of how a system would be used in a real-world setting and how it fits into the work practices of therapists. Furthermore, the prototype relied on mock data because real-life data about upper limb movement over extended periods of time is currently not available. If real-life sensor data were available, it is likely that the data would contain a degree of inaccuracy due to movement of the sensors on the patient’s body and due to sensor drift, which would affect measures of quality and range of motion. Finally, the therapists in this study spoke about the potential uses of the dashboard to engage patients, yet these claims have not been verified with patients. A deployment study of a functioning dashboard and wearable technology with patients engaged in upper limb therapy and their therapists will be conducted in the next phase of this project to address these limitations.

A further limitation of the dashboard and wearable technology developed in this project is the lack of data on wrist and finger extension. The current system focusses on the movement of the arm (shoulder, elbow, and wrist supination/pronation), which is critical for many stroke patients with low levels of mobility. However, activities of daily living like eating, dressing, and washing rely to a great extent on our ability to move the wrist and the fingers, which are not captured in the current design. Related work shows the potential of capturing finger and wrist movements through sensors captured through gloves [33,34] or rings worn on the finger [12,16], which we aim to explore in subsequent phases of this research project.

### Conclusions

Upper limb information from wearable technology provides hitherto unavailable insights into the activities of stroke patients outside the clinic. Visualization of this information provides therapists with objective data, engages patients and supports discussion with other clinicians. Consideration needs to be given to contextual information, such as how to collect this information and how to integrate it with existing visualizations to provide meaningful insights into activities of daily living performed by patients. These findings open the door for further work to develop wearable technology for patients to collect upper limb data in real life, and to develop visualizations that present this information to therapists and patients to support rehabilitation.

## Abbreviations

ADL: activities of daily living
CIMT: constraint-induced movement therapy
IMUs: inertial measurement units
MAL: Motor Activity Log
ROM: range of motion
